# Elementary Microscopical Technology

**Published:** 1888-04

**Authors:** 


ELEMENTARY MICROSCOPICAL TECHNOLOGY. A Manual for Students of Microscopy. Part I. The technical history of a slide from the crude materials to the finished mount. By Frank L. James, M. D., St. Louis. *Medical and Surgical Journal Co. 1887.*

This book is unhesitatingly and earnestly recommended. It will do more practical good in its line and afford more practical instruction than a dozen of the large, costly and "impossible-to-understand-how" treatises. Succinct, simple, condensed and definite, it gives what a beginner always wants—full and minute directions about what to do and how to do it. The excellence of the matter atones largely for the defects of the printing and make-up. It will repay any one interested in microscopy to get it and use it.

---

A LAW DISPENSARY.—There has been established in this city a bureau to which poor people can resort for legal advice free of charge. This bureau was opened under the auspices of reputable lawyers in connection with Rev. Mr. Goss' People's Mission.

There is an opportunity for much good to be done by such a dispensary, and we congratulate the legal fraternity on having joined hands with the medical profession in the organization of philanthropic work.

Law and benevolence are not ordinarily thought to go hand in hand; but no doubt, with the aid of some pastoral appliances, they can be jerked along together for a bit.

We only hope, out of brotherly feeling, that law dispensaries will never grow into such an incubus as medical dispensaries have done, that they will never be used for advertising purposes, or for working up a private business, or for exploiting the good works of the business managers. The medical profession can tell the legal profession a great deal about the lights and shadows of the dispensary system.—*New York Medical Record.*

